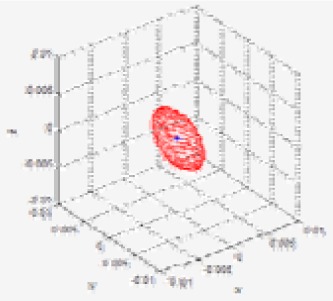# Spatial Uncertainty Model for Visual Features Using a Kinect™ Sensor

**DOI:** 10.3390/s120708640

**Published:** 2012-06-26

**Authors:** Jae-Han Park, Yong-Deuk Shin, Ji-Hun Bae, Moon-Hong Baeg

**Affiliations:** Robot Convergence R & D Group, Korea Institute of Industrial Technology (KITECH), 1271-18, Sa-3-dong, Sangrok-gu, Ansan 426791, Korea; E-Mails: syd@kitech.re.kr (Y.-D.S.); joseph@kitech.re.kr (J.-H.B.); mhbaeg@kitech.re.kr (M.-H.B.)

**Keywords:** Kinect™ sensor, depth sensing camera, 3D acquisition, uncertainty model, visual feature, depth calibration, disparity map, point cloud

## Abstract

This study proposes a mathematical uncertainty model for the spatial measurement of visual features using Kinect™ sensors. This model can provide qualitative and quantitative analysis for the utilization of Kinect™ sensors as 3D perception sensors. In order to achieve this objective, we derived the propagation relationship of the uncertainties between the disparity image space and the real Cartesian space with the mapping function between the two spaces. Using this propagation relationship, we obtained the mathematical model for the covariance matrix of the measurement error, which represents the uncertainty for spatial position of visual features from Kinect™ sensors. In order to derive the quantitative model of spatial uncertainty for visual features, we estimated the covariance matrix in the disparity image space using collected visual feature data. Further, we computed the spatial uncertainty information by applying the covariance matrix in the disparity image space and the calibrated sensor parameters to the proposed mathematical model. This spatial uncertainty model was verified by comparing the uncertainty ellipsoids for spatial covariance matrices and the distribution of scattered matching visual features. We expect that this spatial uncertainty model and its analyses will be useful in various Kinect™ sensor applications.

## 1. Introduction

On 4 November 2010, Kinect™ was launched as a non-contact motion sensing device by Microsoft for the Xbox 360 video game console [1]. However, the remarkable ability of a Kinect™ sensor lies in the important functionality that it can provide after acquisition of high quality 3D scan information in real time at a relatively low cost. Therefore, in addition to motion sensing for gaming, the use of Kinect™ sensors in various applications has been actively investigated in many research areas such as robotics, human-computer interface (HCI), and geospatial information. In the domain of robotics, in particular, many studies are trying to utilize Kinect™ sensors as 3D sensors for perception functionality of intelligent robots [2–7].

Kinect™ sensors provide disparity image and RGB image information simultaneously. Hence, the colored 3D point cloud information could be acquired by fusing the disparity and RGB information from a Kinect™ sensor. However, a calibration process is required for utilizing a Kinect™ sensor as a 3D sensor. For Kinect™ sensor calibration, certain parameters are required: the pin-hole projection and lens distortion parameters of the disparity and RGB cameras, the homogeneous matrix of the two-camera coordinate frame, and the depth calibration parameter, which can transform disparity image data into actual distance. The pin-hole projection and lens distortion parameters of the depth and RGB cameras can be obtained with the existing calibration solution [8,9]. Further, the homogeneous matrix parameters between the depth camera and the RGB camera coordinates can be obtained by the stereo camera calibration method [10,11] or the point cloud matching method [12–14]. Some recent studies have presented results related to depth calibration methods and analyses for acquiring accurate 3D data using the disparity image from a Kinect™ sensor [15–18].

Recently, Kinect™ sensors have been widely utilized as 3D perception sensors in various robotic applications such as 3D mapping, object pose estimation, and Simultaneous Localization and Mapping (SLAM) [3–6]. In these applications, extraction of visual features, matching, and estimation of the 3D position are essential functionalities. Kinect™ sensors are very suitable for these applications because the essential functionalities can be achieved easily using the disparity and the RGB information. These problems can be solved by stochastic optimization methods, which contain measurement error and uncertainties. In this phase, quantitative information about the measurement error and uncertainties of visual features are essential for a reliable estimation result. For example, the covariance matrix of the input noises and errors is the key design parameter for optimal estimation problem using a Kalman filter. In general, all sensors have static and dynamic errors. Static errors, representing the bias of the estimation results, can be corrected by calibration. Dynamic errors, representing the variance of the estimation results, can be improved by filtering methods. However, results for a mathematical uncertainty model representing the covariance matrix form for the spatial measurements of visual features using Kinect™ sensors are unavailable. Khoshelham and Elberink [16] presented an error model and its analysis results; however, these results were represented as an independent error model with respect to the X, Y and Z axis, and not as a covariance matrix. In the Cartesian space, the errors in the X, Y and Z axis data are correlated with each other; thus, the covariance matrix is not in a diagonal form. Therefore, we would like to derive the spatial uncertainty model of visual features using Kinect™ sensors, which is represented by the covariance matrix for 3D measurement errors in the actual Cartesian space.

To achieve this objective, we derive the propagation relationship of the uncertainties between the disparity image space and the real Cartesian space with the mapping function between the two spaces. Then, we obtain the mathematical model for the covariance matrix of the spatial measurement error by using the propagation relationship. Finally, a quantitative analysis of the spatial measurement of Kinect™ sensors is performed by applying the covariance matrix in the disparity image space and the calibrated sensor parameters to the proposed mathematical model.

## 2. 3D Reconstruction from Kinect™ Sensor Data

**Figure 1.**
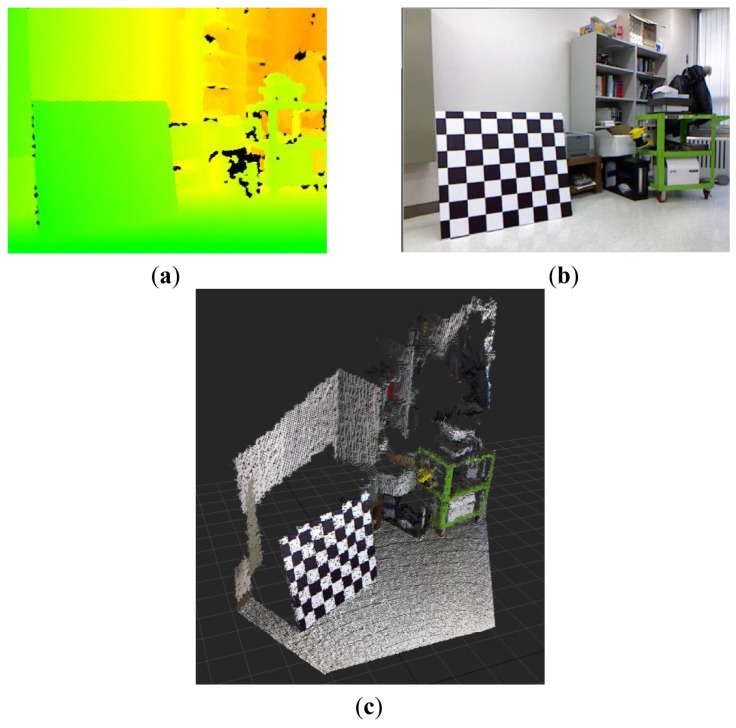
Information from Kinect™ sensor. (**a**) Disparity map image; (**b**) RGB image; (**c**) Colored 3D point cloud data.

**Figure 2.**
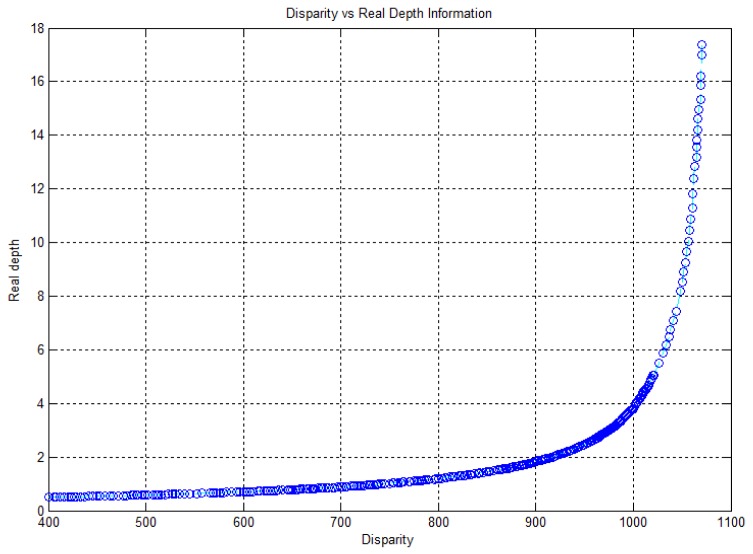
Relationship between the disparity image and the real depth information (disparity: 400–1,069, real depth: 0.5–17.3 m).

Kinect™ sensors provide disparity image and RGB image information. The disparity image represents the spatial information, and the RGB image represents the color information. 3D point cloud data, which contains color information, can be obtained by fusing the disparity image and the RGB image information. Figure 1 shows the disparity image, the RGB image, and the colored 3D point cloud information that was reconstructed from a Kinect™ sensor. Disparity image data, containing information about the distance of the location of each pixel, is expressed as an integer from 0 to 2,047. This data contains relative distance information, which does not represent metric information. In addition, the relationship between distance and disparity image data is non-linear, as shown in the graph in Figure 2. Thus, the depth calibration function, which can transform disparity image data into actual distance information, is needed in order to reconstruct 3D information using Kinect sensors.

The mathematical model between disparity image data *d* and real depth is represented by Equation (1) [16]. In this equation, *Z_o_, f_o_*, and *b* indicate the distance of the reference pattern, focal length, and base length respectively. For depth calibration, two parameters, 1/*Z_o_* and 1/(*f_o_ b*), are determined by the least square fitting method [17]:

(1) $$f(d)=\frac{Z_{o}}{1+\frac{Z_{o}}{f_{o}\cdot b}d}=\frac{1}{\frac{1}{Z_{o}}+\frac{1}{f_{o}\cdot b}\cdot d}$$

In our experiment, the maximum detection range of the Kinect™ sensor was 17.3 m at disparity data 1,069, and the distribution of data changed rapidly beyond a distance of approximately 5 m, as shown in Figure 2. For performing data fitting, the depth calibration model of Equation (1) has only two-degrees-of-freedom for the optimization variables; therefore, it has limitations in representing the curvature of our measurement data. Hence, we proposed an extended depth calibration model using a rational function, which contains higher degree-of-freedom in the optimization variable space [19]. Equation (2) shows the rational function model that is applied to the depth calibration of the Kinect™ sensor:

(2) $$\begin{array}{cc} f(d)=\frac{P(d)}{Q(d)} & \left(P(d)=\sum_{i=1}^{m}\theta_{i}d^{i-1},Q(d)=\sum_{i=1}^{n}\theta_{m+i}d^{i-1}\right) \end{array}$$

where *P*(*d*) is the numerator polynomial and *Q*(*d*) is the denominator polynomial.

**Figure 3.**
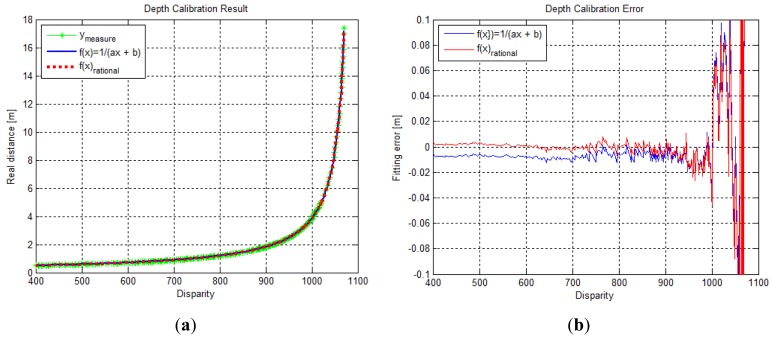
Depth calibration results. (**a**) Fitting results (measurement data, Equation (1) model, fourth-order rational function model); (**b**) Residual (fitting error) results (Equation (1) model, fourth-order rational function model).

To perform depth calibration with the rational function model, a non-linear optimization method such as the Levenberg-Marquardt algorithm can be used. We obtained the depth calibration function with fourth-order polynomials of the numerator and denominator, which can transform disparity data into a real distance of up to approximately 15 m. The depth calibration parameters for the fourth-order rational function model are shown in Table 1. Figure 3 shows the fitting results and the fitting residual results for the depth calibration function in Equations (1) and (2), respectively. In Figure 3(a), both calibration functions seemed to fit the measurement data well. However, as seen in Figure 3(b), the residual error of the rational function model appeared to be nearer to the X-axis than the model represented by Equation (1). This implies that the rational function model with a higher degree-of-freedom of the optimization variables can be fitted more precisely in the depth calibration problem. The norm of residual vector for Equation (1) and the rational function were computed to be 1.045495 and 1.034060, respectively.

After performing depth calibration, the disparity image data can be transformed into the actual distance information by the depth calibration function. Using this actual distance information, the 3D spatial position information can be reconstructed with the pin-hole camera projection model. Equation (3) shows the mapping relationship between the disparity image space data **u** = [*u v* d]*^T^* and the spatial position information **x** = [x y z]*^T^* in the Cartesian space. *u* and *v* are the horizontal and vertical coordinates, respectively, of the disparity image, and *d* is the disparity data, expressed as an integer from 0 to 2,047. *f*(*d*) is the actual distance information that is calculated by the depth calibration function:

(3) $$\begin{array}{c} \text{x}=\text{F}(\text{u})\\ \left[\begin{array}{c} x\\ y\\ z \end{array}\right]=f(d)\left[\begin{array}{ccc} \frac{1}{f_{\text{Depth},x}} & 0 & -\frac{C_{\text{Depth},x}}{f_{\text{Depth},x}}\\ 0 & \frac{1}{f_{\text{Depth},y}} & -\frac{C_{\text{Depth},y}}{f_{\text{Depth},y}}\\ 0 & 0 & 1 \end{array}\right] \end{array}\left[\begin{array}{c} u\\ v\\ 1 \end{array}\right]$$

In the pin-hole camera projection model, *f_Depth,x_* and *f_Depth,y_* are focal length parameters, while *C_Depth,x_* and *C_Depth,y_* are optical axis parameters of the depth camera. These parameters can be obtained using various general camera calibration methods. The pin-hole camera projection parameters are shown in Table 2. We obtained these parameters using the Matlab camera calibration toolbox developed by Bouguet [20].

## 3. Spatial Uncertainty Model of Kinect™ Sensor

The disparity image data from Kinect™ sensors can be converted into the 3D spatial point cloud data using the depth calibration function and the pin-hole camera projection model. However, the 3D position information of the visual features using Kinect™ sensors contains some errors caused by various sources such as inaccurate measurement of disparity, lighting condition, properties of the object surfaces in the disparity data, and image processing and matching errors in the image coordinates. In order to utilize sensor data in actual applications, the information about reliability or uncertainty of the sensor is very important. In this study, we would like to propose a mathematical model for the 3D measurement information, which can provide qualitative and quantitative analysis for Kinect™ sensors.

### 3.1. Qualitative Analysis of Spatial Uncertainty

**Figure 4.**
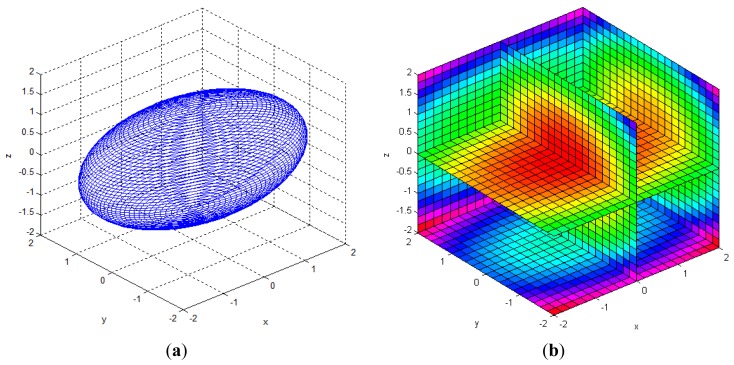
Visualization of a 3D Gaussian model. (**a**) Uncertainty ellipsoid for (**x** – **m***_x_*)*^T^***Q**^–1^(**x** – **m***_x_*) = *k*. (**b**) Density of p.d.f. *g*(**x**). where $\left({\text{m}}_{x}=\left[\begin{array}{c} 0\\ 0\\ 0 \end{array}\right]\;,\;\text{Q}=\left[\begin{array}{ccc} 1.0^{2} & 0 & 0\\ 0 & 0.6^{2} & 0\\ 0 & 0 & 0.5^{2} \end{array}\right]\right)$.

The reliability of the measured 3D information can be represented by the multi-dimensional Gaussian model in the Cartesian space, as shown in Equation (4) and Figure 4. In the Gaussian model, random variables are modeled using the mean vector and the covariance matrix. The error of the mean vector with respect to the measured data is estimation bias, which should be corrected by calibration. The variance parameter of the Gaussian model represents uncertainties of the measurements, and it can be represented as an uncertainty ellipsoid related to the covariance matrix. Thus, we tried to derive a mathematical model of the covariance matrix that describes the spatial uncertainties:

(4) $$\begin{array}{c} g_{\text{xyz}}(\text{x})={\left(2\pi \right)}^{-3}{\left|\text{Q}\right|}^{-\frac{1}{2}}\exp\left\{-\frac{1}{2}{\left(\text{x}-{\text{m}}_{x}\right)}^{T}{\text{Q}}^{-1}\left(\text{x}-{\text{m}}_{x}\right)\right\}\\ \left(\text{x}={\left[x,y,z\right]}^{T},{\text{m}}_{x}={\left[m_{x},m_{y},m_{z}\right]}^{T}\right) \end{array}$$

**Figure 5.**
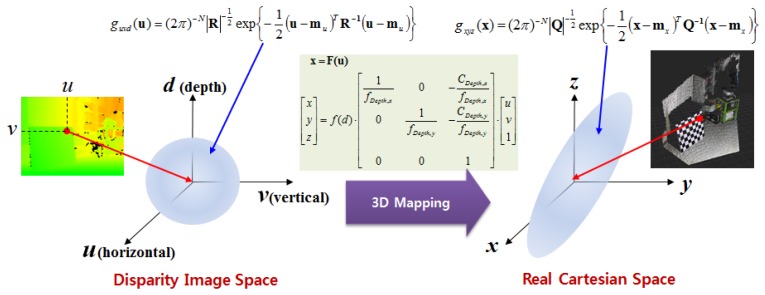
Mapping relationship of uncertainty between the disparity image space and the real Cartesian space.

To derive the spatial uncertainty model, the mapping relationship between the disparity image space and the real Cartesian space should be considered. Figure 5 shows this mapping relationship. Owing to the absence of correlations between the elements of vector **u** in the disparity image space, the covariance matrix **R** of vector **u** has a diagonal form, as shown in Equation (5). This deduction can be confirmed from experimental data. The symbols σ*_u_* and σ*_v_* represent the variance corresponding to the visual feature position image co-ordinates *u* and *v*, respectively. The variance is caused by image processing errors such as image pixel quantization and key point localization. The symbol σ*_d_* represents the variance of disparity measurements, which result from inaccuracy, lighting condition, and properties of the object surfaces [16]. Thus, the elements of vector [*u, v, d*]*^T^* are unrelated, and the diagonal elements of the covariance matrix can be obtained independently. In addition, the causes of errors are independent of vector **u**, and hence, the covariance matrix **R** can be assumed to be the same in the entire disparity image space:

(5) $$\text{R}=\left[\begin{array}{ccc} \sigma_{u}^{2} & 0 & 0\\ 0 & \sigma_{v}^{2} & 0\\ 0 & 0 & \sigma_{d}^{2} \end{array}\right]$$

Uncertainty in the actual space appears as the propagation of uncertainty in the disparity image space by mapping relations. If the relationship between the two spaces is a linear mapping such as **y** = **Ax**, the propagated output covariance matrix **Q** is determined as Q = **ARA***^T^* for the input covariance matrix **R** [21]. However, as shown in Equation (3), the relationship between the two spaces is a non-linear mapping. Therefore, we can obtain the covariance matrix in the actual space by a linearized approximation of the mapping function using Jacobian matrix, as shown in Equation (6).

(6) $$\begin{array}{c} \text{J}(\text{u})=\frac{\partial \text{F}(\text{u})}{\partial \text{u}}=\left[\begin{array}{ccc} \frac{f(d)}{f_{\text{Depth},x}} & 0 & \frac{\partial f(d)}{\partial d}\left(\frac{u}{f_{\text{Depth},x}}-\frac{C_{\text{Depth},x}}{f_{\text{Depth},x}}\right)\\ 0 & \frac{f(d)}{f_{\text{Depth},y}} & \frac{\partial f(d)}{\partial d}\left(\frac{v}{f_{\text{Depth},y}}-\frac{C_{\text{Depth},y}}{f_{\text{Depth},y}}\right)\\ 0 & 0 & \frac{\partial f(d)}{\partial d} \end{array}\right]\\ f(d)=\frac{P(d)}{Q(d)}\;,\;\frac{\partial f(d)}{\partial d}=\frac{\dot{P}(d)Q(d)-P(d)\dot{Q}(d)}{Q{\left(d\right)}^{2}} \end{array}$$

Thus, we can obtain the mathematical model of spatial uncertainty shown in Equation (7), and the uncertainty ellipsoid for Kinect™ measurement can be estimated in the entire measurable space using this covariance matrix model:

(7) $$\begin{array}{c} \text{Q}=\text{J}(\text{u})\cdot \text{R}\cdot \text{J}{\left(\text{u}\right)}^{T}\\ \text{Q}=\left[\begin{array}{ccc} \sigma_{u}^{2}A^{2}+\sigma_{d}^{2}C^{2}D^{2} & \sigma_{d}^{2}C^{2}DE & \sigma_{d}^{2}C^{2}D\\ \sigma_{d}^{2}C^{2}DE & \sigma_{v}^{2}B^{2}+\sigma_{d}^{2}C^{2}E^{2} & \sigma_{d}^{2}C^{2}E\\ \sigma_{d}^{2}C^{2}D & \sigma_{d}^{2}C^{2}E & \sigma_{d}^{2}C^{2} \end{array}\right] \end{array}$$

where $\left(\begin{array}{l} A=\frac{f(d)}{f_{\text{Depth},x}},\;B=\frac{f(d)}{f_{\text{Depth},y}},\;C=\frac{\partial f(d)}{\partial d},\;D=\frac{u-C_{\text{Depth},x}}{f_{\text{Depth},x}},\;E=\frac{v-C_{\text{Depth},y}}{f_{\text{Depth},y}}\\ {\text{J}}_{\left(u,v,d\right)}=\left[\begin{array}{ccc} A & 0 & C\cdot D\\ 0 & B & C\cdot E\\ 0 & 0 & C \end{array}\right],\;\text{R}=\left[\begin{array}{ccc} \sigma_{u}^{2} & 0 & 0\\ 0 & \sigma_{v}^{2} & 0\\ 0 & 0 & \sigma_{d}^{2} \end{array}\right] \end{array}\right)$

### 3.2. Quantitative Analysis of Spatial Uncertainty

In order to perform a quantitative analysis of the uncertainty model, quantitative data obtained from the real sensor is needed. Hence, the actual sensor parameters such as the depth calibration parameters, pin-hole projection parameters of the depth camera, and covariance matrix information in the disparity image space are required. The depth calibration parameter and the pin-hole camera parameters are shown in Tables 1 and 2, respectively. The diagonal elements of covariance matrix **R** (σ*_u_*, σ*_v_*, and σ*_d_*) are obtained from the disparity image data of visual features by tracking 850 feature points in the real experimental environment. The SURF [22] algorithm was used to detect and match visual features for tracking the trajectory of feature points. The variances σ*_u_*, σ*_v_*, and σ*_d_* were estimated as 1.051, 0.801, and 1.266, respectively, from experimental data. Equation (8) represents the Jacobian matrix and the input covariance matrix **R**, which was obtained from real sensor parameters and experimental data:

(8) $$\text{J}(\text{u})=\left[\begin{array}{ccc} 0.0017\cdot f(d) & 0 & \frac{\partial f(d)}{\partial d}\left(0.0017\cdot u-0.549\right)\\ 0 & 0.0017\cdot f(d) & \frac{\partial f(d)}{\partial d}\left(0.0017\cdot v-0.443\right)\\ 0 & 0 & \frac{\partial f(d)}{\partial d} \end{array}\right]\;,\;\text{R}=\left[\begin{array}{ccc} 1.051^{2} & 0 & 0\\ 0 & 0.801^{2} & 0\\ 0 & 0 & 1.266^{2} \end{array}\right]$$

**Figure 6.**
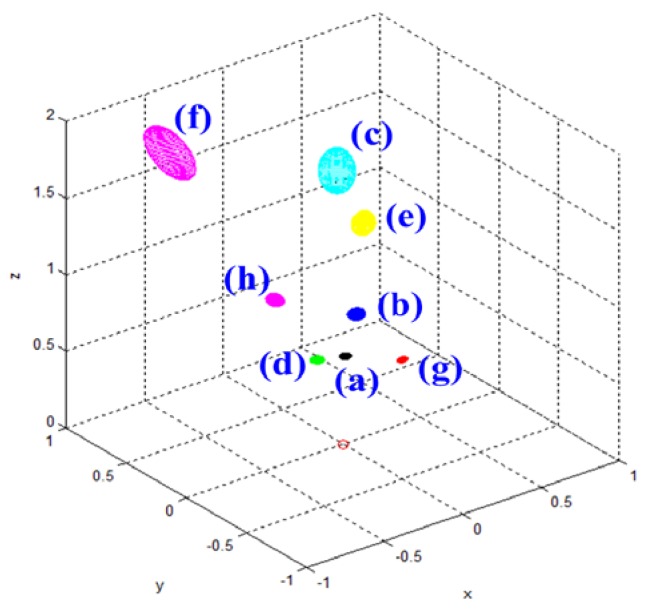
Uncertainty ellipsoids for all test cases.

Table 3 shows the Gaussian parameters (mean vector and covariance matrix), square root value of covariance matrix norm (maximum standard deviation), vector of maximum direction, and the uncertainty ellipsoid, for cases (a)–(h). The symbol ε in the covariance matrix represents a very small number with a near-zero value. From the results in various cases, it is observed that the spatial uncertainties vary with the distance and the image coordinates of the measurement position. Figure 6 shows all the uncertainty ellipsoids for cases (a)–(h) in the Cartesian space. From the test cases, it can be seen that the volume and direction of the uncertainty ellipsoids are closely related to the measurement position.

**Figure 7.**
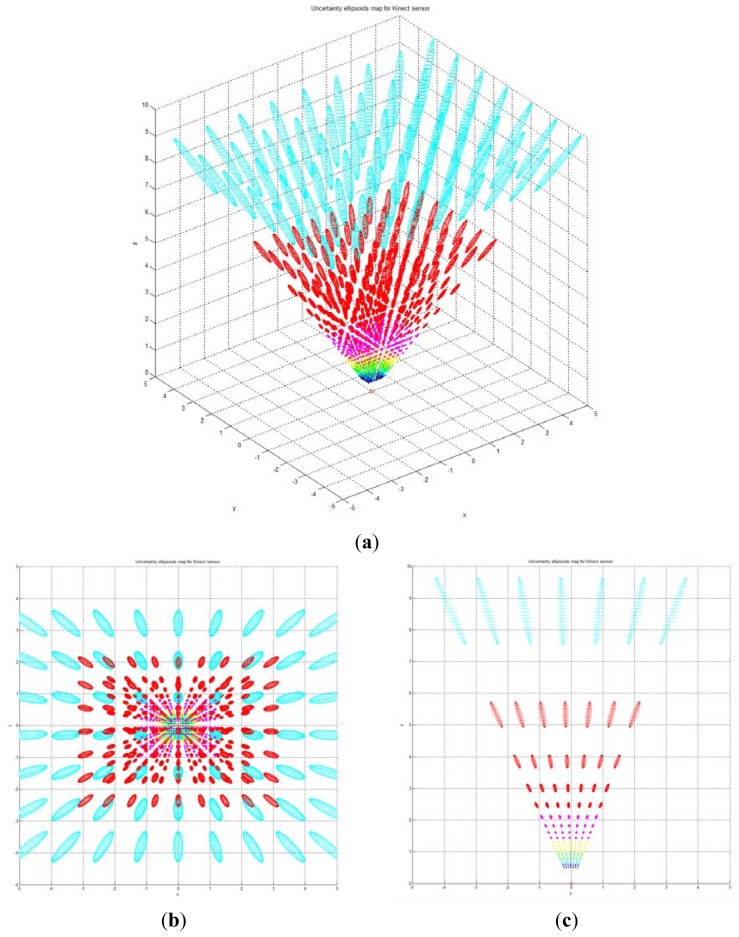
(**a**) Uncertainty ellipsoid map in the entire measurable Cartesian space; (**b**) View of *x-y* plane direction; (**c**) View of *y-z* plane direction.

Figure 7 shows the uncertainty ellipsoid map for the Kinect™ sensor in the entire measurable Cartesian space. This uncertainty map is constructed by drawing the 3D ellipsoid for **x***^T^***Q**^–1^**x** = *k*, and calculating each spatial covariance matrix Q by using Equation (7) with increment steps of 40 for *u, v*, and *d*. The uncertainty ellipsoid map represents the distribution in volume and direction of the longest axis of uncertainty ellipsoids in the entire space. From this uncertainty map, it can be concluded that the volume of the uncertainty ellipsoid is greatly influenced by the distance of the measured point and its maximum direction is related to the direction of the optical axis of the sensor.

**Figure 8.**
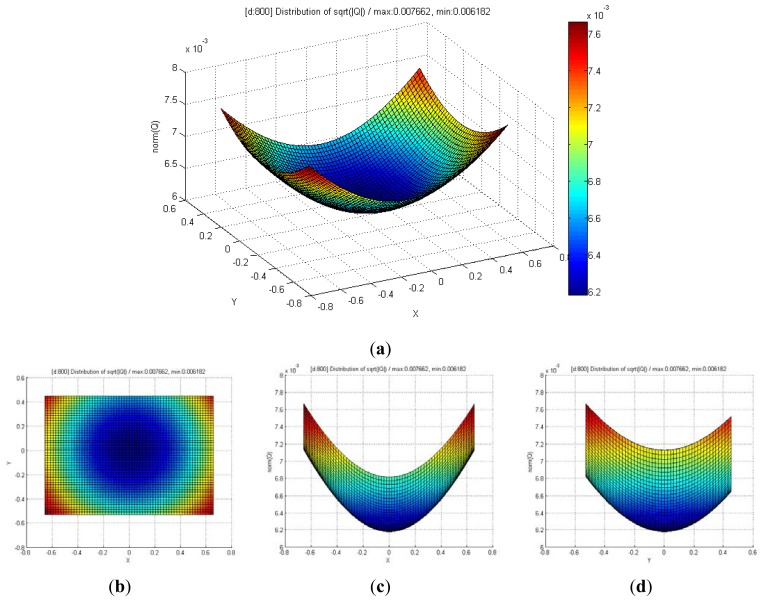
(**a**) Distribution of maximum standard deviation for various values of *u* and *v*, and fixed value of *d* (*d* = 800, *real distance* = 1.2 m); (**b**) View of *x-y* direction; (**c**) View of *x-z* direction; (**d**) View of *y-z* direction.

**Figure 9.**
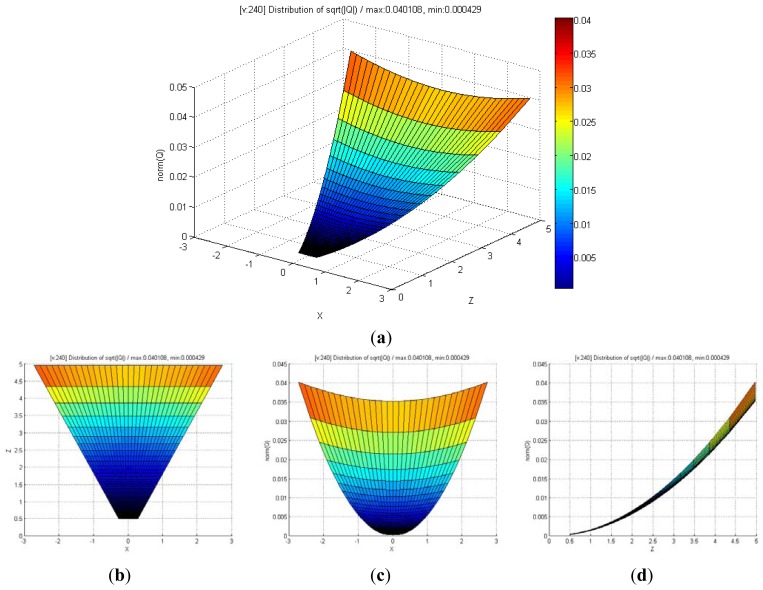
(**a**) Distribution of maximum standard deviation for various values of *u* and *d*, and fixed value of *v* (*v* = 240, Vertical center of disparity image); (**b**) View of *x-y* direction; (**c**) View of *x-z* direction; (**d**) View of *y-z* direction.

**Figure 10.**
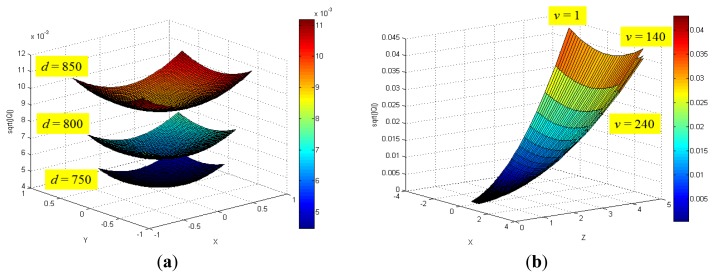
(**a**) Integrated distribution of the maximum standard deviation for various values of *u* and *v*, and three values of *d* (750, 800, 850); (**b**) Integrated distribution of the maximum standard deviation for various values of *u* and *d*, and three values of *v* (1, 140, 240).

**Figure 11.**
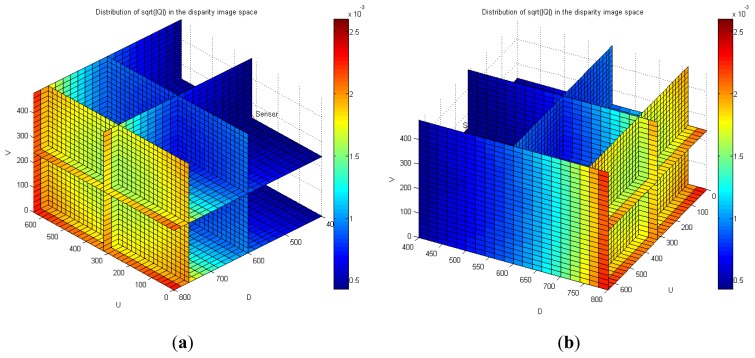
Volume distribution of the maximum standard deviation for most of the disparity image space. (**a**) View of (AZ = –135°, EL = 45°); (**b**) View of (AZ = 120°, EL = 45°).

Figure 8 shows the distribution of maximum standard deviation (square root of norm for the covariance matrix **Q**) of the spatial uncertainties by varying *u* and *v*, and by keeping *d* fixed. The results showed a quadratic distribution in the Cartesian space when the depth remains the same. The measurement point is farther from the center of the optical axis in the image coordinate, and hence, the maximum standard deviation attains a higher value. Figure 9 shows the distribution of the maximum standard deviation obtained by varying *u* and *d*, and by keeping *v* fixed. Its distribution resembled a fan type plane in the Cartesian space when the horizontal measure remains the same. From the distribution, it can be observed that the maximum standard deviation increases with an increase in the depth. Further, an increase in depth causes a steeper gradient because it is farther from the center of the image coordinate. Figure 10 shows the integrated distribution of the maximum standard deviation for (a) various values of *u* and *v*, and three values of *d* and (b) various values of *u* and *d*, and three values of *v*. Further, Figure 11 shows the volume distribution of the maximum standard deviation for most of the disparity image space. From these analyses, it can be confirmed that spatial uncertainty varies with the distance and the image coordinates of the measurement position.

## 4. Experiments and Results

### 4.1. Estimation of the Input Covariance Matrix **R**

**Figure 12.**
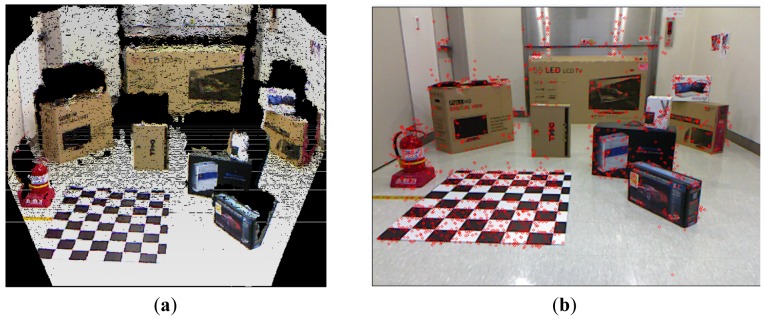
Experimental environment. (**a**) Colored point cloud representation; (**b**) Detected SURF visual features.

**Figure 13.**
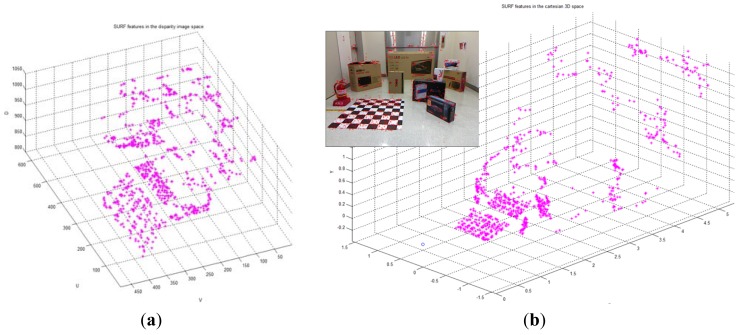
Detected visual features in the experimental environment. (**a**) View of the disparity image space; (**b**) View of the real Cartesian space.

**Figure 14.**
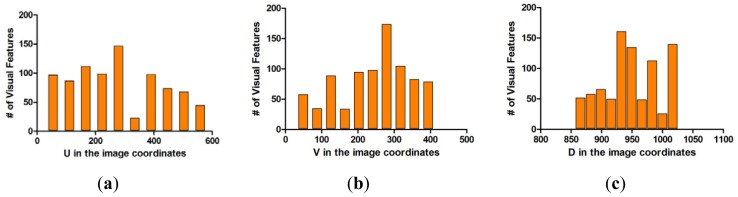
Histogram distribution of detected visual features in the disparity image space. (**a**) *u* data of visual features. (**b**) *v* data of visual features. (**c**) *d* data of visual features.

The covariance matrix in the disparity image is necessary for the calculation of the spatial uncertainty model. We tried to estimate the input covariance matrix **R** in the disparity image space from real experiments. Figure 12 shows the overall experimental environment for estimation of the matrix **R**. In this experimental environment, objects were placed at various locations and orientations in order to obtain visual feature information with a uniform distribution at various conditions and in the entire measurable space. Figure 12(b) shows the visual feature detection results obtained by using the SURF algorithm in this experimental environment. As shown in Figure 12(b), 850 visual features were obtained. Each visual feature is tracked continuously by the SURF matching function, and the corresponding trajectory information is recorded. The index of each visual feature is assigned randomly during the first detection phase. Figure 13(a,b) shows the distribution of the 850 detected visual features in the disparity image space and the real Cartesian space, respectively. Figure 14 shows a histogram representation of the distribution of the visual features in the image space with respect to the *u, v*, and *d* axes. The distribution of the histogram for *u, v*, and *d* axes confirmed that the visual features were uniformly distributed in the entire image space.

Each visual feature was obtained from 100 data measurements by matching and tracking, and the mean **m***_i_* and the covariance matrix **R***_i_* in Equation (9) were calculated from the measurement data. The covariance matrices were calculated differently owing to various reasons. However, the mean covariance matrix was computed in order to characterize the representative covariance matrix. Based on Equation (10), the mean covariance matrix was computed from the covariance matrix of 850 visual features.

**Figure 15.**
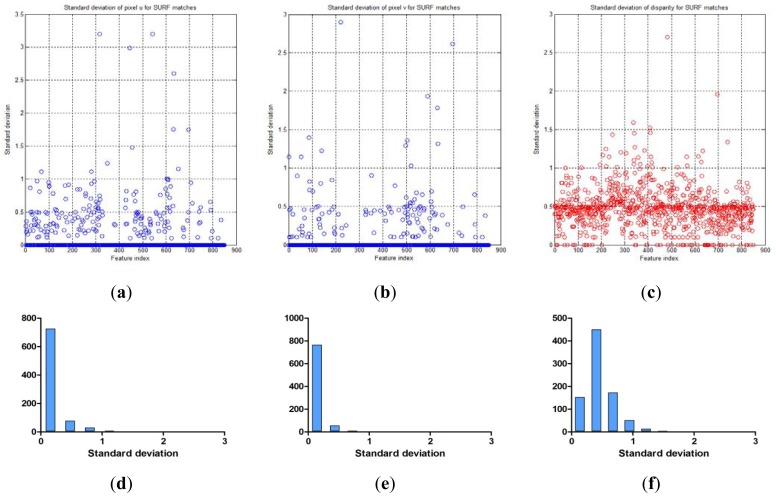
Standard deviations of visual features and histogram; (**a**) Standard deviations for *u* of visual features; (**b**) Standard deviations for *v* of visual features; (**c**) Standard deviations for *d* of visual features; (**d**) Histogram distribution for (**a**); (**e**) Histogram distribution for (**b**); (**f**) Histogram distribution for (**c**).

**Figure 16.**
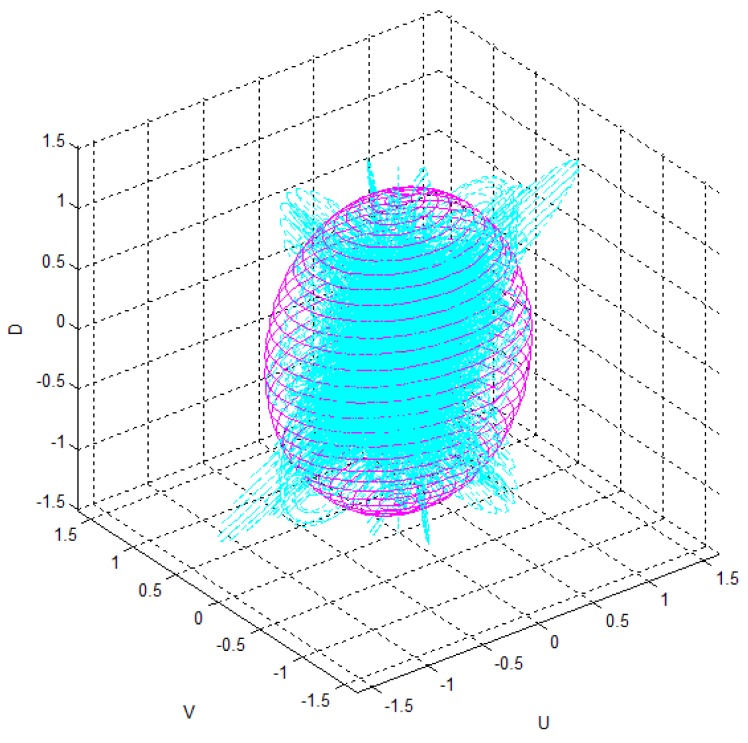
Uncertainty ellipsoids for all the visual features and the estimated covariance matrix in the disparity image space. (Cyan: ellipsoids for covariance matrix **R***_i_*, Magenta: ellipsoid for the estimated covariance matrix **R**).

It was confirmed that the mean covariance matrix in Equation (10) was similar to the diagonal matrix form. Thus, the covariance matrix in the disparity image space can be assumed to be a diagonal matrix as in Equation (5), and the diagonal elements of the covariance matrix can be computed independently. Figure 11 shows the standard deviation data and its histogram for each of the 850 visual features, corresponding to the values of *u, v*, and *d*. As shown in Figure 15, the deviations for visual features were observed as random variables. Further, the statistical parameters should be obtained by taking most of the element data into consideration. From the data in Figure 15, the mean of standard deviation for u-axis and its deviation were 0.118 and 0.311, respectively. The mean of standard deviation for v-axis and its deviation were 0.072 and 0.243, respectively. The mean of standard deviation for d-axis and its deviation were 0.477 and 0.263, respectively. The input covariance matrix **R** should be determined by utilizing most of the covariance matrix **R***_i_* for each feature. Hence, the 3σ level threshold (99.7%), which can include most of the covariance matrices, was used for determining the elements of the covariance matrix **R**, as shown in Equation (11). Therefore, the estimated covariance matrix **R** represents the statistically worst case of measurement at the 3σ level. Figure 16 shows the uncertainty ellipsoids for all the visual features and the estimated covariance matrix in the disparity image space. From the result, it can be confirmed that the ellipsoid for the estimated covariance matrix includes most of the ellipsoids for the visual features:

(9) $$\begin{array}{c} {\text{m}}_{i}=\frac{1}{100}\sum_{k=1}^{100}{\text{u}}_{i,k}=\frac{1}{100}\sum_{k=1}^{100}\;{\left[\begin{array}{c} u_{i}\\ v_{i}\\ d_{i} \end{array}\right]}_{k},\;{\text{R}}_{i}=\frac{1}{100}\sum_{k=1}^{100}\left[\left({\text{u}}_{i,k}-{\text{m}}_{i}\right){\left({\text{u}}_{i,k}-{\text{m}}_{i}\right)}^{T}\right]\\ \left(\text{i}:\text{Feature index}\;1\sim 850\right) \end{array}$$

(10) $$\bar{\text{R}}=\frac{1}{850}\sum_{i=1}^{850}{\text{R}}_{i}=\left[\begin{array}{ccc} 0.1564 & 0.0107 & -0.0145\\ 0.0107 & 0.1099 & -0.0153\\ -0.0145 & -0.0153 & 0.4761 \end{array}\right]$$

(11) $$\begin{array}{c} \sigma_{u}=\text{mean}\left(\sigma_{u}(i)\right)+3\cdot \text{dev}\left(\sigma_{u}(i)\right)=1.051\\ \sigma_{v}=\text{mean}\left(\sigma_{v}(i)\right)+3\cdot \text{dev}\left(\sigma_{v}(i)\right)=0.801\\ \sigma_{d}=\text{mean}\left(\sigma_{d}(i)\right)+3\cdot \text{dev}\left(\sigma_{d}(i)\right)=1.266 \end{array}\;,\;\text{R}=\left[\begin{array}{ccc} 1.051^{2} & 0 & 0\\ 0 & 0.801^{2} & 0\\ 0 & 0 & 1.266^{2} \end{array}\right]$$

### 4.2. Comparison with the Uncertainty Model and the Distribution of Real Visual Features

**Figure 17.**
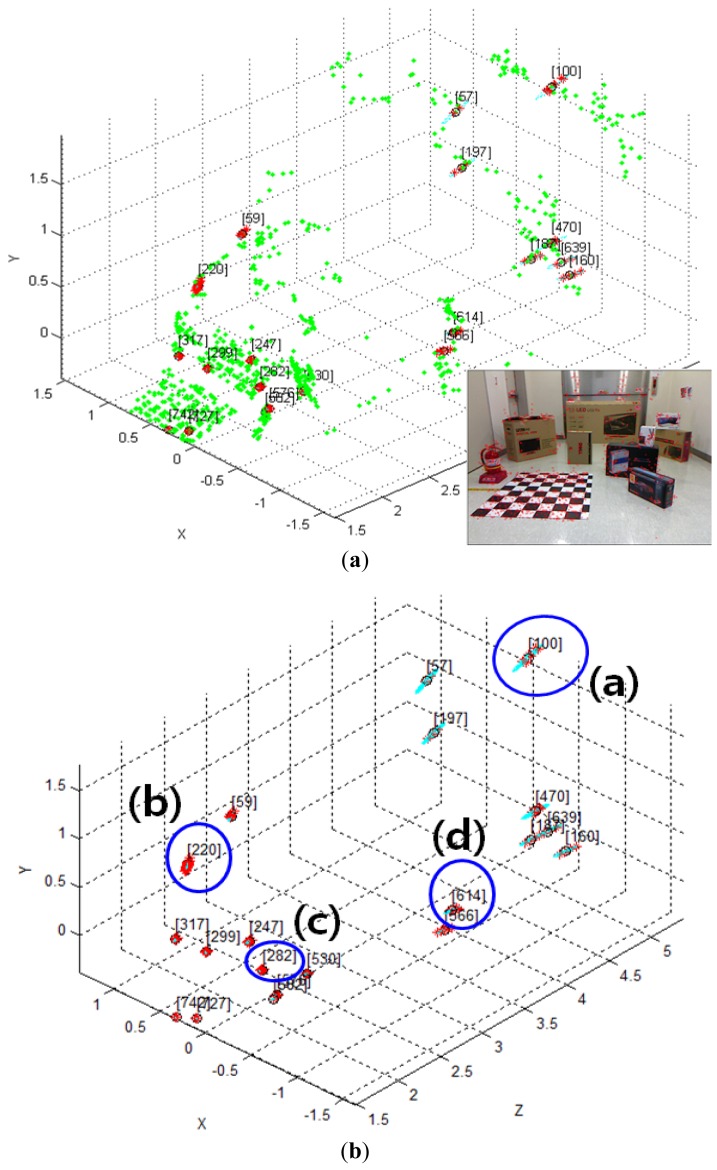
20 representative visual features in the Cartesian space. (**a**) 20 representative visual features highlighted among all the visual features; (**b**) 20 representative visual features and their uncertainty ellipsoids.

**Figure 18.**
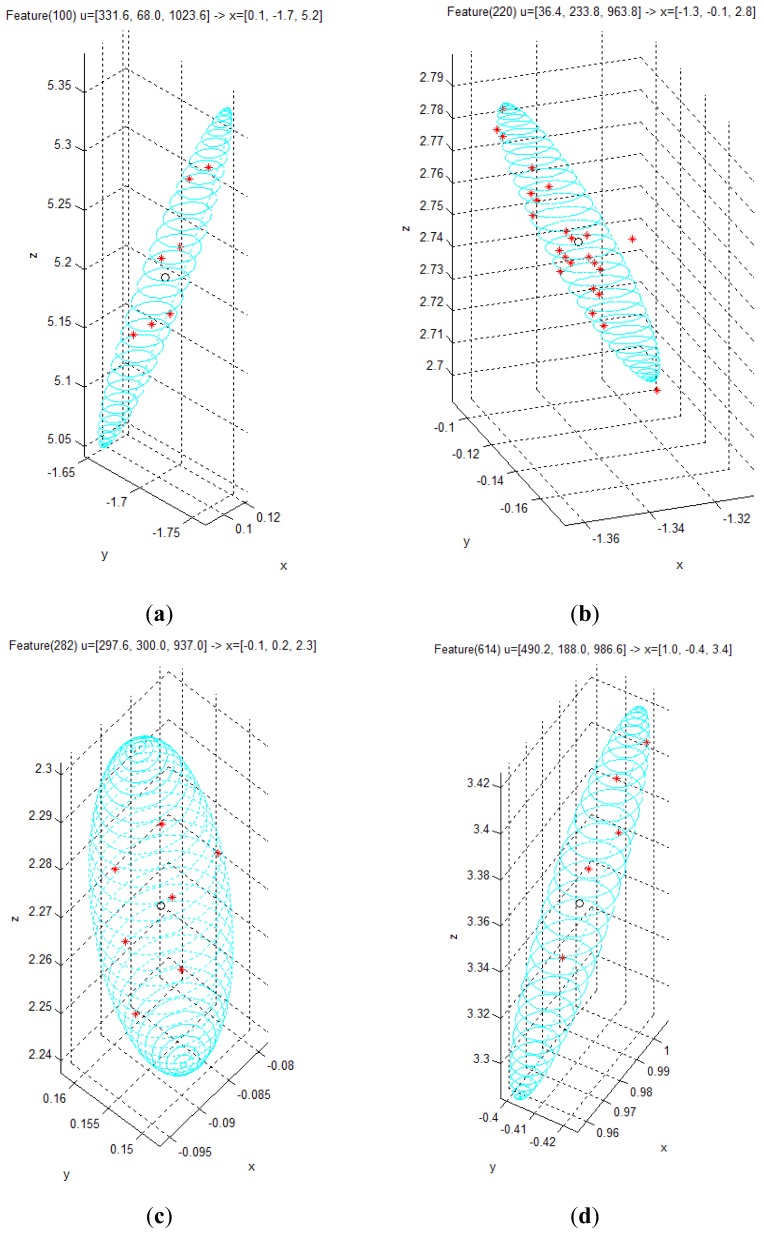
Distribution of 3D measurements for 4 visual features and their uncertainty ellipsoids. (**a**) id: 100; (**b**) id: 220; (**c**) id: 282; (**b**) id: 614.

Given the covariance matrix in the disparity image space and the Kinect™ calibration parameters, the spatial covariance matrix can be calculated by using the uncertainty model proposed in this study. Using this spatial uncertainty model, we drew the uncertainty ellipsoid map shown in Figure 7, and we could identify its shape, volume, and direction in the Cartesian space. However, it must be confirmed that the calculated uncertainty model can represent the distribution of scattered measurement data. This can be verified by comparing the uncertainty ellipsoid with the distribution of the 3D position for tracked visual features. To confirm that the uncertainty model met all the requirements, some representative features were selected from the 850 visual features, and the uncertainty ellipsoid and the distribution of tracked measurement for the visual features were compared. Figure 17 shows the 20 selected visual features highlighted among all the visual features. As shown in Figure 17(a), the representative visual features were selected to ensure maximum possible uniform distribution in the Cartesian space. Figure 17(b) shows the 3D measurements for the visual features and the calculated uncertainty matrices, represented by the red symbol (*) and the cyan ellipsoids, respectively, for 20 visual features in one frame. However, owing to the difficulties in representing the scale corresponding to each feature, Figure 17(b) is not suitable for performing a detailed analysis. Hence, the results of features (a)–(d) in Figure 17(b) were represented again in Figure 18 by modifying the scale to obtain more detailed results. Then, the uncertainty ellipsoid was compared with the distribution for 3D measurement of visual features.

**Figure 19.**
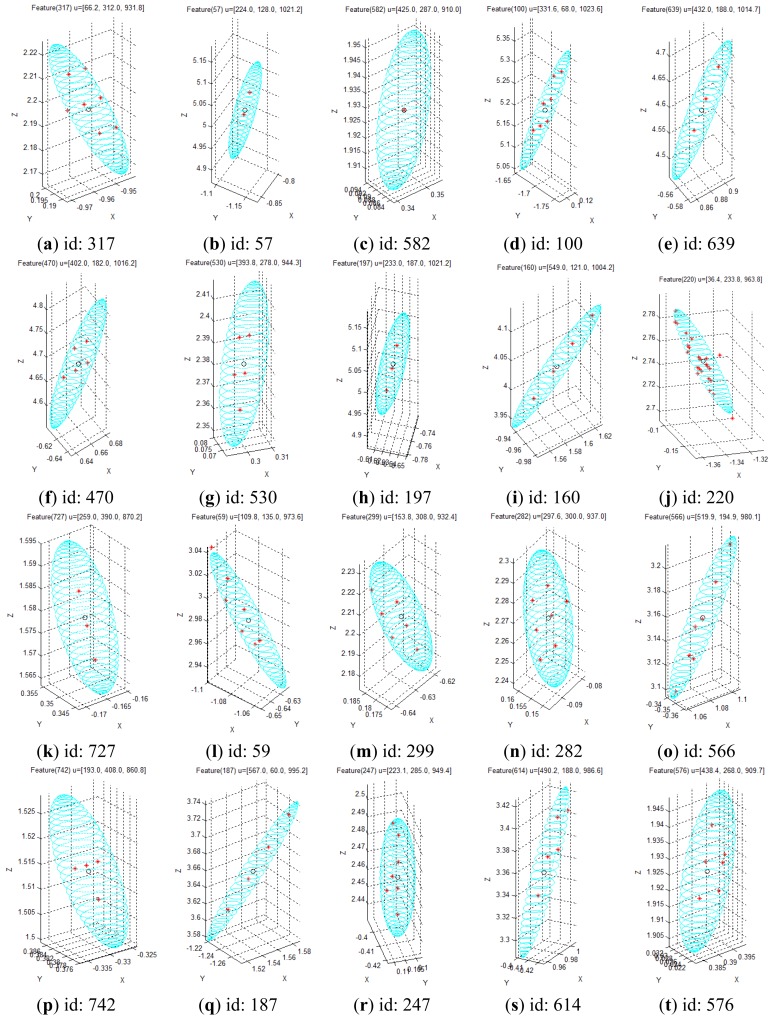
Distribution of 3D measurements for the 20 selected visual features.

Figure 18(a) shows the results for the visual feature with id “100”, acquired at **u** = [331.6, 68, 1,023.6]*^T^* in the disparity image space. In the result, the measurements represented by the red symbol (*) appear at seven points clustered around the point **x** = [0.1, –1.7, 5.2]*^T^* in the Cartesian space. The input data in the disparity image space is discrete, and hence, the transformed 3D measurement must also be discrete. Therefore, the 3D measurement distribution is observed as a discrete distribution, and 100 measurements for this visual feature overlap at seven points. Further, it is verified that the cyan uncertainty ellipsoid includes all the 3D measurements corresponding to the visual feature. Figure 18(b) shows the results for the visual feature with id “220”, acquired at **u** = [36.4, 233.8, 963.8]*^T^* in the disparity image space. In the result, the symbols representing the measurements are seen at various points clustered around the point **x** = [–1.3, –0.1, 2.8]*^T^* in the Cartesian space. Further, the uncertainty ellipsoid includes most of the 3D measurements corresponding to the visual feature. In this distribution, 3 measurements are located slightly outside the ellipsoid boundary, but their distances from the boundary are extremely small. Figure 18(c) shows the results for the visual feature with id “282”, acquired at **u** = [297.6, 300.0, 937.0]*^T^* in the disparity image space. In the result, the measurements appear at 7 points clustered around the point **x** = [–0.1, 0.2, 2.3]*^T^* in the Cartesian space, and the uncertainty ellipsoid includes all the 3D measurements corresponding to the visual feature. Figure 18(d) shows the results for the visual feature with id “614”, acquired at **u** = [490.2, 188.0, 986.6]*^T^* in the disparity image space. In the result, the measurements appear at 5 points clustered around the point **x** = [1.0, –0.4, 3.4]*^T^* in the Cartesian space, and the uncertainty ellipsoid includes all the 3D measurements corresponding to the visual feature. The results for all the 20 visual features are represented in Figure 19 by modifying the scale to obtain more detailed results. The overall results show that the simple equation for the proposed spatial uncertainty model represents the worst case model for image space uncertainties; however, it was confirmed that the spatial uncertainty model provided a sufficiently good description of the discrete distribution for most of the 3D measurements of the visual features.

## 5. Conclusions

In this study, we proposed a mathematical model for spatial measurement uncertainty, which can provide qualitative and quantitative analysis for Kinect™ sensors. To achieve this objective, we derived the spatial covariance matrix model using the mapping function between the disparity image space and the actual Cartesian space. Next, we performed a quantitative analysis of the spatial measurement errors using actual sensor parameters. In order to derive the quantitative model of the spatial uncertainty for the visual features, we estimated the covariance matrix in the disparity image space using the collected visual feature data. Further, we computed the spatial uncertainty information by applying the covariance matrix in the disparity image space and the calibrated sensor parameters to the proposed mathematical model. This spatial uncertainty model was verified by comparing the uncertainty ellipsoids for spatial covariance matrices and the distribution of scattered matching visual features. Quantitative analysis of a Kinect™ sensor facilitates the availability of concrete information about the sensor, rather than abstract information. For example, abstract information, such as “If the measurement distance increases, the uncertainty will be increased”, could be transformed into concrete information, such as “Maximum error at a measurement distance of 1.2 m is 1.68 cm at the level 3σ.”

Recently, Kinect™ sensors have been widely utilized as 3D perception sensors for intelligent robots to solve various problems such as 3D mapping, object pose estimation, and SLAM. In these actual applications, information about the reliability and the uncertainty of the visual features for 3D measurements is very important. Hence, we expect that the uncertainty model presented in this paper will be useful in many applications that employ Kinect™ sensors.

## Figures and Tables

**Table 1.** Depth calibration parameters (4th-order rational function model).

**Polynomial order**	**Const. (*d*^0^)**	**1st (*d*^1^)**	**2nd (*d*^2^)**	**3rd (*d*^3^)**	**4th (*d*^4^)**
Numerator *P*(*d*)	452.705	−611.068	255.254	−7.295	7.346
Denominator *Q*(*d*)	−326.149	588.446	−548.754	340.178	−47.175

**Table 2.** Pin-hole camera projection parameters of the depth camera.

**Parameter**	*f_Depth,x_*	*f_Depth,y_*	*C_Depth,x_*	*C_Depth,y_*
Value	582.64	586.97	320.17	260.00

**Table 3.** Covariance matrix **Q**, maximum deviation and maximum vector, uncertainty ellipsoids.

Test case	u	x (mx) (mean)	Q (Covariance matrix)	$\sqrt{{\left\|\text{Q}\right\|}_{2}}$ (max. deviation)	v (max. vector)	Uncertainty ellipsoid **x***^T^* **Q^−1^x** = *k*
(a)	$\left[\begin{array}{c} 320\\ 240\\ 500 \end{array}\right]$	$\left[\begin{array}{c} -0.0002\\ -0.0199\\ 0.5837 \end{array}\right]$	$10^{-5}\times \left[\begin{array}{ccc} 0.1109 & \varepsilon & -\varepsilon \\ \varepsilon & 0.0637 & -0.0057\\ -\varepsilon & -0.0629 & 0.1684 \end{array}\right]$	0.0013	$\left[\begin{array}{c} -0.0008\\ -0.0545\\ 0.9985 \end{array}\right]$	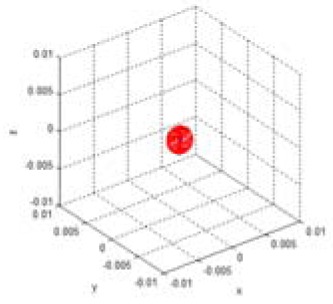
(b)	$\left[\begin{array}{c} 330\\ 200\\ 700 \end{array}\right]$	$\left[\begin{array}{c} 0.0149\\ -0.0906\\ 0.8861 \end{array}\right]$	$10^{-5}\times \left[\begin{array}{ccc} 0.2557 & -0.0014 & 0.0139\\ -0.0014 & 0.1548 & -0.0842\\ 0.0139 & -0.0842 & 0.8236 \end{array}\right]$	0.0029	$\left[\begin{array}{c} 0.0241\\ -0.1230\\ 0.9921 \end{array}\right]$	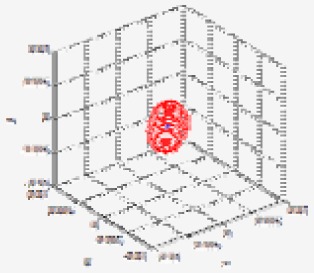
(c)	$\left[\begin{array}{c} 300\\ 250\\ 900 \end{array}\right]$	$\left[\begin{array}{c} -0.0631\\ -0.0311\\ 1.8227 \end{array}\right]$	$10^{-3}\times \left[\begin{array}{ccc} 0.0110 & 0.0001 & -0.0052\\ 0.0001 & 0.0062 & -0.0025\\ -0.0052 & -0.0025 & 0.1491 \end{array}\right]$	0.0122	$\left[\begin{array}{c} -0.0373\\ -0.0178\\ 0.9991 \end{array}\right]$	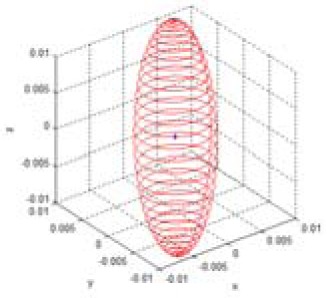
(d)	$\left[\begin{array}{c} 100\\ 150\\ 600 \end{array}\right]$	$\left[\begin{array}{c} -0.2666\\ -0.1322\\ 0.7054 \end{array}\right]$	$10^{-5}\times \left[\begin{array}{ccc} 0.2098 & 0.0238 & -0.1268\\ 0.0238 & 0.1044 & -0.0629\\ -0.1268 & -0.0629 & 0.3354 \end{array}\right]$	0.0021	$\left[\begin{array}{c} -0.5091\\ -0.2003\\ 0.8371 \end{array}\right]$	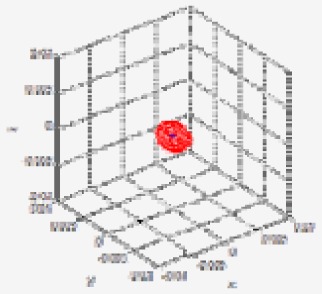
(e)	$\left[\begin{array}{c} 490\\ 400\\ 800 \end{array}\right]$	$\left[\begin{array}{c} 0.3474\\ 0.2842\\ 1.1917 \end{array}\right]$	$10^{-4}\times \left[\begin{array}{ccc} 0.0693 & 0.0189 & 0.0793\\ 0.0189 & 0.0419 & 0.0649\\ 0.0793 & 0.0649 & 0.2722 \end{array}\right]$	0.0056	$\left[\begin{array}{c} -0.3137\\ -0.2392\\ -0.9189 \end{array}\right]$	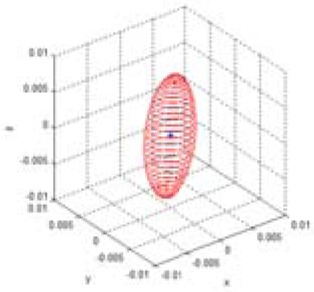
(f)	$\left[\begin{array}{c} 80\\ 360\\ 920 \end{array}\right]$	$\left[\begin{array}{c} -0.8402\\ 0.3473\\ 2.0383 \end{array}\right]$	$\begin{array}{l} 10^{-3}\times \\ \left[\begin{array}{ccc} 0.0531 & -0.0163 & -0.0959\\ -0.0163 & 0.0145 & 0.0397\\ -0.0959 & 0.0397 & 0.2327 \end{array}\right] \end{array}$	0.0168	$\left[\begin{array}{c} -0.3923\\ 0.1587\\ 0.9060 \end{array}\right]$	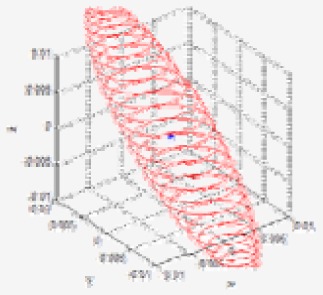
(g)	$\left[\begin{array}{c} 600\\ 80\\ 450 \end{array}\right]$	$\left[\begin{array}{c} 0.2577\\ -0.1646\\ 0.5366 \end{array}\right]$	$10^{-5}\times \left[\begin{array}{ccc} 0.1209 & -0.0174 & 0.0566\\ -0.0174 & 0.0647 & -0.0361\\ 0.0566 & -0.0361 & 0.1178 \end{array}\right]$	0.0014	$\left[\begin{array}{c} -0.6613\\ 0.2960\\ -0.6893 \end{array}\right]$	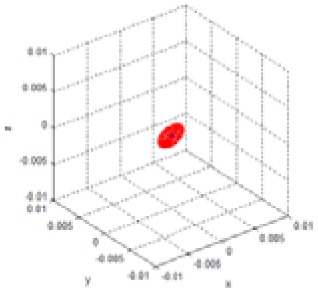
(h)	$\left[\begin{array}{c} 180\\ 450\\ 700 \end{array}\right]$	$\left[\begin{array}{c} -0.2132\\ 0.2868\\ 0.8861 \end{array}\right]$	$10^{-5}\times \left[\begin{array}{ccc} 0.2557 & -0.0014 & 0.0139\\ -0.0014 & 0.1548 & -0.0842\\ 0.0139 & -0.0842 & 0.8236 \end{array}\right]$	0.0031	$\left[\begin{array}{c} -0.2902\\ 0.3398\\ 0.8946 \end{array}\right]$